# Nutraceutical Evaluation of Trigonelline's Therapeutic Potential by Targeting Bladder Cancer Stem Cells and Cancer-Associated Fibroblasts via Downregulation of TGFβ3/GLI2/YAP1 Signaling Hub

**DOI:** 10.7150/ijms.107228

**Published:** 2025-02-18

**Authors:** Chien-Chang Kao, Jing-Wen Shih, Huong Thi Luu Kim Huynh, Ching-Hsin Chang, Bashir Lawal, Sitthichai Iamsaard, Nur Azizah, Ritmaleni Ritmaleni, Justin Kung-Yi Lin, Po-Yang Huang, Alexander T.H. Wu, Ming-Che Liu

**Affiliations:** 1 Division of Urology, Department of Surgery, Tri-Service General Hospital, National Defense Medical Center, Taipei, Taiwan.; 2 Ph.D. Program for Cancer Molecular Biology and Drug Discovery, College of Medical Science and Technology, Taipei Medical University and Academia Sinica, Taipei 11031, Taiwan.; 3 Graduate Institute of Cancer Biology and Drug Discovery, College of Medical Science and Technology, Taipei Medical University, Taipei 11031, Taiwan.; 4 TMU Research Center of Cancer Translational Medicine, Taipei Medical University, Taipei 11031, Taiwan.; 5 International PhD Program for Translational Science, College of Medical Science and Technology, Taipei Medical University, Taipei 11031, Taiwan.; 6 Institute of Microbiology and Immunology, National Yang-Ming Chiao Tung University, Hsinchu 30010, Taiwan.; 7 Institute of Microbiology and Immunology, National Yang-Ming University, Taipei, 11221, Taiwan.; 8 TMU Research Center of Urology and Kidney, Taipei Medical University, Taipei, 11031, Taiwan.; 9 UPMC Hillman Cancer Center, University of Pittsburgh, USA.; 10 Department of Anatomy, Faculty of Medicine, Khon Kaen University, Khon Kaen 40002, Thailand.; 11 Research Institute for Human High Performance and Health Promotion, Khon Kaen University, Khon Kaen 40002, Thailand.; 12 Laboratory of Medicinal Chemistry, Department of Pharmaceutical Chemistry, Faculty of Pharmacy, Universitas Gadjah Mada, Jl. Sekip Utara,Yogyakarta, 55281, Indonesia.; 13 Curcumin Research Center, Faculty of Pharmacy, Universitas Gadjah Mada, Jl. Sekip Utara, Yogyakarta, 55281, Indonesia.; 14 SPs Traditional Medical Clinic, Taipei, Taiwan.; 15 The Ph.D. Program of Translational Medicine, College of Medical Science and Technology, Taipei Medical University, Taipei, 11031, Taiwan.; 16 Clinical Research Center, Taipei Medical University Hospital, Taipei Medical University, Taipei 11031, Taiwan.; 17 Graduate Institute of Medical Sciences, National Defense Medical Center, Taipei 11490, Taiwan.; 18 Taipei Heart Institute, Taipei Medical University, Taipei 11031, Taiwan.; 19 School of Dental Technology, College of Oral Medicine, Taipei Medical University Taipei 11031, Taiwan.; 20 Department of Urology, Taipei Medical University Hospital Taipei 11031, Taiwan.; 21 Graduate Institute of Clinical Medicine, School of Medicine, College of Medicine, Taipei Medical University Taipei 11031, Taiwan.

**Keywords:** trigonelline, bladder cancer, cancer stem cells, cisplatin resistance, cancer-associated fibroblasts, TGFβ3/GLI2/YAP1 signaling

## Abstract

Trigonelline (TGN), an alkaloid identified in medicinal plants such as coffee (Coffea spp.) and fenugreek (Trigonella foenum-graecum), has demonstrated significant anticancer properties across various malignancies, yet its efficacy in bladder cancer (BLCA) remains underappreciated. This study investigates TGN's role in modulating cancer stem cells (CSCs) and the tumor microenvironment (TME), two key contributors to BLCA progression and chemoresistance. Through comprehensive bioinformatics analyses of BLCA patient datasets, a TGY signature (TGFβ3, GLI2, YAP1) was identified as a critical signaling hub associated with poor prognosis, therapeutic resistance, and CSC generation. Computational docking studies revealed TGN's high binding affinity to the TGY signature, TGFβ3 (ΔG = -3.9 kcal/mol), GLI2 (ΔG = -4.2 kcal/mol), YAP1 (ΔG = -3.4 kcal/mol), suggesting its potential to disrupt this signaling axis. *In vitro* experiments demonstrated that TGN effectively inhibited BLCA cell proliferation, colony formation, and tumorspheroid growth while significantly enhancing cisplatin sensitivity in resistant cell lines. Notably, TGN reduced the transformation of fibroblasts into cancer-associated fibroblasts (CAFs) through the downregulation of α-SMA and FAP (Fibroblast activation protein) expression, indicating its capacity to normalize the TME. Real-time PCR analysis revealed that TGN treatment significantly reduced markers of epithelial-mesenchymal transition and stemness pathways. Our preclinical mouse study demonstrated that combining TGN and cisplatin significantly reduced tumorigenesis in cisplatin-resistant bladder tumoroids harboring CAFs. Importantly, this combination therapy showed no apparent systematic toxicity, suggesting a favorable safety profile. Our findings reveal novel molecular targets of TGN in bladder cancer; TGN acts as a potent disruptor of the TGY signaling axis and a normalizer of the TME by reducing CAF transformation. In sum, our findings advocate for TGN's further exploration as a candidate for combination therapy in drug-resistant BLCA, with the potential to improve patient outcomes by simultaneously targeting both CSCs and the TME, serving as a foundation for future clinical trials.

## Introduction

Bladder cancer (BLCA) is a complex urinary tract malignancy and the 5th most common cancer globally, with a low survival rate 1. BLCA ranks the highest fatality worldwide, with over 400,000 new cases reported annually 2. The treatment and five-year survival rates have remained the same for more than three decades; this necessitates the urgency for novel diagnostic and therapeutic interventions for bladder cancer 3. Recently, immunotherapy has shown promising anti-tumor effects in BLCA 4. The tumor microenvironment (TME) significantly advances BLCA progression and consequently affects patients' prognosis. Since the infiltration of immune/stromal cells with the TME can significantly alter the gene expression and behaviors of cancer cells, identifying the signaling pathways that connect the tumor cells and TME can offer improved clinical outcomes in cancer patients 5, 6.

Both experimental and clinical evidence strongly indicated that the immune/stromal cell lineages within the tumor microenvironment are essential determinants of tumor progression or regression 7. The major cellular components include cancer-associated fibroblasts (CAFs), M2-subtype tumor-associated macrophages (TAMs), and regulatory T (Treg) cells, all of which have been implicated in promoting metastasis and poor clinical outcomes in bladder cancer 8. In advanced cancer, the Transforming Growth Factor-β (TGFβ) signaling pathway plays a significant role in suppressing the immune system mainly through deregulation of cytokine production as well as inactivating T-cell cytotoxic properties 9, 10. Also, TGFβs are multifunctional cytokines excreted by the cancer cells and immune cells and can regulate the immune system 11. The TGFβ family comprises four different isoforms, TGFβ1, TGFβ2, TGFβ3, and TGFβ4; their primary functions are involved in cellular processes, including cell proliferation/apoptosis and metastasis (especially in the epithelial-mesenchymal transition (EMT) process) 12, 13. Embryonically, TGFβ3 activates Lef1, bypassing β-catenin via nuclear phosphorylated Smad2 and Smad4 and promotes epithelial-to-mesenchymal transition (EMT) 14. In a pan-cancer sequencing analysis, TGFβ signaling was predominantly linked to the EMT and stemness in the bladder cancer cohort 15. Our preliminary research from the TCGA bladder cancer database revealed that patients with high expression of TGFβ3 were significantly associated with a lower overall survival rate than patients with a lower TGFβ3 expression. Notably, TGFβ3 is a less researched member of the TGFβ family, and emerging evidence indicates TGFβ3 signaling promotes EMT and cell mobility in cancer cells 16. Thus, this prompted us to clarify the role of TGFβ3-associated signaling in bladder carcinogenesis.

Cancer stem cells (CSCs) reside within the heterogeneity of tumors and participate in repopulating the tumor mass after chemo- and targeted therapies, resulting in treatment failure 17-19. Studies have indicated that the tumor microenvironment is pivotal in promoting the generation and survival of CSCs 20-22. CAFs are a major component of the tumor microenvironment and have been shown to contribute to tumorigenesis and cancer stemness; they are involved in various aspects of cancer progression, including facilitating tumor survival, angiogenesis, invasion, distant metastasis, and chemoresistance 23. Based on these premises, we examined TGFβ-associated signaling networks in bladder CSC generation and its link to CAF transformation.

Coffee is a widely consumed beverage, well-known for its caffeine content. However, the complex biochemical makeup of coffee goes beyond just caffeine. One of its other significant components is an alkaloid known as trigonelline (TGN). In high concentrations in raw coffee beans, TGN is a subject of considerable interest due to its transformation and potential health benefits. TGN undergoes a series of chemical transformations during the roasting process. These changes lead to the formation of various compounds, including N-methylpyridinium iodide (NMP) and its related picolinium analogs. This transformation is particularly noteworthy because of the potential health implications of these compounds. TGN has been shown to inhibit the Nrf2-mediated anti-apoptotic pathway, thereby overcoming etoposide resistance in pancreatic cancer cells 24. Notably, Nrf2 is the downstream effector of the PI3K/Akt signaling pathway where Nrf2 functions as a cytoprotective factor regulating the expression of genes coding for antioxidant, anti-inflammatory, and detoxifying proteins (hence drug-resistance) 25. Enhanced NRF2-NPC1L1 signaling was recently demonstrated to play a significant role in drug-tolerant cancer cells 26. Notably, elevated NRF2 and YAP1 expressions have been shown to promote chemoresistance in bladder cancer 27, suggesting that TGN could potentially target YAP1, a member of the TGY signature identified bioinformatically. Also, TGN has been demonstrated to have anticancer functions through inhibiting PI3K, Akt, and PLCγ signaling. Given its inhibitory effects on Nrf2, PI3K/AKT signaling, and potentially YAP1, TGN is a therapeutic candidate for drug-resistant bladder cancer.

Furthermore, our preliminary computational docking analysis revealed that TGN could bind to TGF-β3 with a high affinity. These interactions suggest that TGN could directly target the key components of the TGY signature, making it a promising candidate for overcoming cisplatin resistance in bladder cancer and improving therapeutic outcomes. Here, we unveiled new therapeutic targets of TGN, TGFβ3-associated signaling in cisplatin-resistant bladder cancer cells.

## Materials and Methods

### Pan-cancer transcriptomic profiling revealed GLI2 and TGFβ3 as key molecular links to EMT/CSC signatures in bladder cancer

Through data mining, we found a seminal study that broadly demonstrated the correlations of GLI1/2 with TGFβ, which uncovered the crucial role of TGFβ as the supporter in the progression of cancers with elevated GLI1/2. Notably, the pan-cancer dataset provided insight into the correlations between these target genes and EMT/stemness signature 28. Consequently, we reanalyzed the data and focused on the bladder cancer cohort. The Pearson correlation was utilized for mutual correlation analysis between genes in TGY signature, while a multivariate linear model was applied to explore the correlation between TGY signatures and EMT/stemness signatures. GraphPad Prism version 9.5.0 for Windows, GraphPad Software, San Diego, California, USA, illustrated the heatmap of these correlations. To investigate the potential pathways in which TGFβ3 might be involved, we utilized the Harmonizome 3.0 to acquire the 42 TGFβ3-associated genes from Pathway Commons Protein-Protein Interactions 29. The SRplot was used to visualize the KEGG pathway of these gene sets 30.

### Identification of TGFβ3/GLI2/YAP1 as the theragnostic signature in the BLCA cohort

The Kaplan-Meier plotter is a web-based tool that provides the survival time of patients from various cancers from the TGCA database 31. Therefore, we used this valuable tool to investigate the association between the overall survival (OS) rate of BLCA patients and the expression of each gene in the TGY signature. The cutoff value identified the lower and upper quartiles of gene expression, and Cox regression analysis was used to evaluate the survival rate.

To further enhance the crucial role of the TGY signature, the association of combined expression of TGFβ3/GLI2/YAP1 and the overall survival outcome of BLCA patients was explored by using the GEPIA computational method 32. The BLCA cohort was split into high and low TGY expression groups based on the median cutoff from the Mantel-Cox test.

The risk of individual or combined expression in TGY signature was considered statistically significant when the hazard ratio (HR) > 1 and p-value of < 0.05.

### Investigation of the correlation of TGY signature and the infiltrated immune cells and CAFs

TIMER2.0 is a powerful website for exploring the correlation between query genes and immune cell infiltration 33. Therefore, to explore the association between TGFβ3/GLI2/YAP1 and immune cells and CAFs, we used this computational method, which was measured by Spearman correlation. A significant correlation was identified with p-value < 0.05.

To investigate the gene expression of the TGY signature associated with T-cell exclusion phenotypes, such as CAFs, MDSCs and M2, we used TIDE, the website that provided information on T-cell dysfunction characteristics and immunotherapy resistance concerning gene set patterns 34. Furthermore, TIDE utilized the huge transcriptomic data from various cancers, especially the dataset Mariathasan2018_PDL1 - metastatic urothelial carcinoma (mUC, n = 348 patients). The OS rate of mUC patients was also evaluated regarding TGFβ3/GLI2/YAP1 expression, the Cox-Proportional Hazards regression was used for these survival analyses.

### Drugs and chemicals

TGN was purchased from Selleckchem (HPLC: 99.98% purity, Cat No. S3220, Taipei, Taiwan). A 10 mM stock solution of TGN was prepared in dimethyl sulfoxide (DMSO) and kept frozen at a temperature of -20 °C. Cell culture media was obtained from Thermo Fisher Scientific, Inc. Waltham, MA, USA). Other reagents and chemicals were purchased from Sigma Aldrich (St. Louis, MO, USA) unless otherwise specified.

### Cell viability assay

Approximately 3×10^3^ T24 and 5637 cells per well of 96-well plates were incubated overnight in DMEM medium. Cells were treated with varying concentrations of TGN or cisplatin for 48 hs. Cell viability was then assayed using established SRB reagent protocols 35.

### Cell culture and isolation of CD44^+^ cells

Human bladder cancer cell lines T24 and 5637 were purchased from ATCC and cultured and maintained under the suggested conditions. CD44^+^ T24 and 5637 cells were identified and isolated using fluorescence-activated cell sorting (FACS).

### Tumorspheroid and tumoroid generation

The generation of bladder tumorspheroids was performed following a previously established protocol with slight modifications 36. Briefly, 1 million cells per 6-well plate were incubated in DMEM medium with 5% CS-FBS at 37°C in a humidified CO_2_ incubator for 24 hs. Subsequently, the cells were transferred to ultra-low-attachment 6-well plates (Corning Inc., Taipei, Taiwan) at a concentration of 20,000 cells per well in DMEM/f12 medium (Gibco, CA, USA) and supplemented with insulin epidermal growth factor (20 ng/ml), basic fibroblast growth factor (10 ng/ml), N2 and B27. The supplements were all purchased from Invitrogen (CA, USA). Tumorspheroids were visualized and quantified using a phase-contrast microscope (Leica, Taipei, Taiwan). Tumoroids were generated by co-culturing bladder tumorspheroids and normal fibroblasts (100,000 cells, ATCC, USA) as described above.

### Gene-silencing experiments

T24 and 5637 cells were transfected as per the protocol described previously 37 with 20 nM of scrambled (siCtrl) or siRNA (siTGFβ3) obtained from OriGene Technologies Inc. (cat#: SR321124). Transfected cells were harvested after 48 hs and processed for subsequent analysis.

### RNA isolation and real-time polymerase chain reaction (PCR)

Total RNA was isolated and purified by using TRIzol-reagent (Life Technologies). Five hundred nanograms (500 ng) of total RNA was reverse-transcribed (RT) using a Qiagen OneStep RT-PCR Kit (Qiagen, New Taipei City, Taiwan), and the PCR was performed using a Rotor-Gene SYBR Green PCR Kit (400, Qiagen). Primers used for the quantitative (q)PCR experiments are listed in Supplementary Data (**Table S1**).

### Western blot analysis

Cell samples were harvested and prepared for Western blot analysis following different treatments (drugs or siRNA transfection). Total protein lysates from cells were separated by 10% sodium dodecyl sulfate-polyacrylamide gel electrophoresis (SDS-PAGE), transferred onto polyvinylidene difluoride membranes (Bio-Rad Laboratories, Hercules, CA, USA), and blocked (with 5% skimmed milk) for non-specific binding. Finally, the membrane was immunoblotted with primary antibodies (Supplementary Data, Table S2) overnight at 4°C and secondary antibodies (horseradish peroxidase-linked anti-rabbit and mouse, 1:5000) for 1-2 hs at room temperature. Target protein signals from the membrane were detected with an enhanced chemiluminescence kit (Thermo Fisher Scientific, Waltham, MA, USA).

### Determination of CAFs infiltration and IL-6 secretion

Tumoroids were fixed and permeabilized with a methanol-acetone mixture for 15 minutes. For blocking non-specific binding, 5% BSA in PBS was used. For the characterization of CAFs, an anti-α-SMA (1:200, Cat # A2547 mouse monoclonal, clone 1A4, Merck, Taipei, Taiwan) antibody was utilized. A goat anti-mouse IgG (H+L) cross-adsorbed secondary antibody, Alexa Fluor™ 594 (1:2000), was used to visualize CAFs. The immunofluorescence imaging was conducted using an Olympus IX83 fluorescent microscope and CellSens Imaging Software (Olympus Life Science, Waltham, MA, USA). The extent of CAF infiltration was determined by the immunofluorescence compared to their control.

IL-6 secretion was determined using a dot plot protocol from a previous study 38. In brief, culture media from control and drug-treated tumoroids were collected and concentrated using an ultra-centrifugal filter tube (Cat# UFC8010, 10 kDa MWCO, Merk, Taipei, Taiwan). The total protein in the medium concentrates was determined using a BCA Protein Assay Kit (Cat# A55865, Thermo Scientific, Taipei, Taiwan). An equal amount of total protein from each sample was plotted on a PVDF membrane. IL-6 was then detected using anti-IL-6 antibody as a standard western blot protocol 39. The intensity of immunoreaction of IL-6 in each sample was determined using ImageJ software.

### Molecular docking simulations

In order to explore if TGN could target the TYG gene signature, we performed a molecular docking simulation. The three-dimensional (3D) predicted structures of TGF-β3, YAP1, and GLI2 (in PDB format) were retrieved from the RCSB Protein Data Bank 40, corresponding to PDB IDs 1TGJ, 3KYS, and 7RX0, respectively. The 3D structures of TGN (CID 5570) and dioxane (CID 31275) were obtained in SDF format from PubChem (https://pubchem.ncbi.nlm.nih.gov/) 41. Subsequently, the SDF files were converted to PDB format by PyMOL software 42, followed by conversion to PDBQT format using AutoDock, which involved adding hydrogen atoms, removing water molecules, and assigning Kollman charges. The interactions between TGN, dioxane, and the proteins TGF-β3, YAP1, and GLI2 were then examined using AutoDock. The grid spacing was set to 1 Å, with dimensions of 40 × 40 × 40 Å along the x, y, and z axes. PyMOL was used to visualize the 3D ligand-receptor complexes. At the same time, the 2D (two-dimensional) interaction diagrams were generated and analyzed using BIOVIA Discovery Studio 43.

### *In vivo* study

The animal studies were carried out according to the regulations of the institutional animal care and use committee, Taipei Medical University (approval number: LAC2022-0405). NOD/SCID female mice (7 weeks old) were purchased from BioLASCO (Taiwan). The 5637 tumorspheroids containing CAFs were disassociated and subcutaneously implanted into the right flank of the animal (10^6^ cells per injection). Mice were then grouped into vehicle control (n=5) and 3 treatment groups (n=5 each): TGN monotherapy (50 mg/kg, 3 times/week, i.p), cisplatin monotherapy (1 mg/kg, 3 times/week, i.p), and combination therapy (TGN: 50 mg/kg, 3 times/week, i.p; cisplatin: 1 mg/kg, 5 times/week, i.p). The tumor size was measured using a standard caliper using the formula: volume V = ½ (Length × Width^2^). Animals' weights were measured weekly. After the experiments, tumor samples were harvested and processed for subsequent analyses.

### Statistical analysis

Experiments were conducted in triplicates, and data were analyzed using the GraphPad Prism version 6.04 for Windows, GraphPad Software (La Jolla, California, USA). Results are expressed as mean ± SD of assays performed 3 times in triplicate. The students' t-tests were performed to compare the statistical results within the groups. *p < 0.05, **p < 0.01, ***p < 0.001.

## Results

### Identification of TGFβ3/GLI2/YAP1 signaling as a potential therapeutic gene signature for drug-resistant bladder cancer

**Figure 1:**
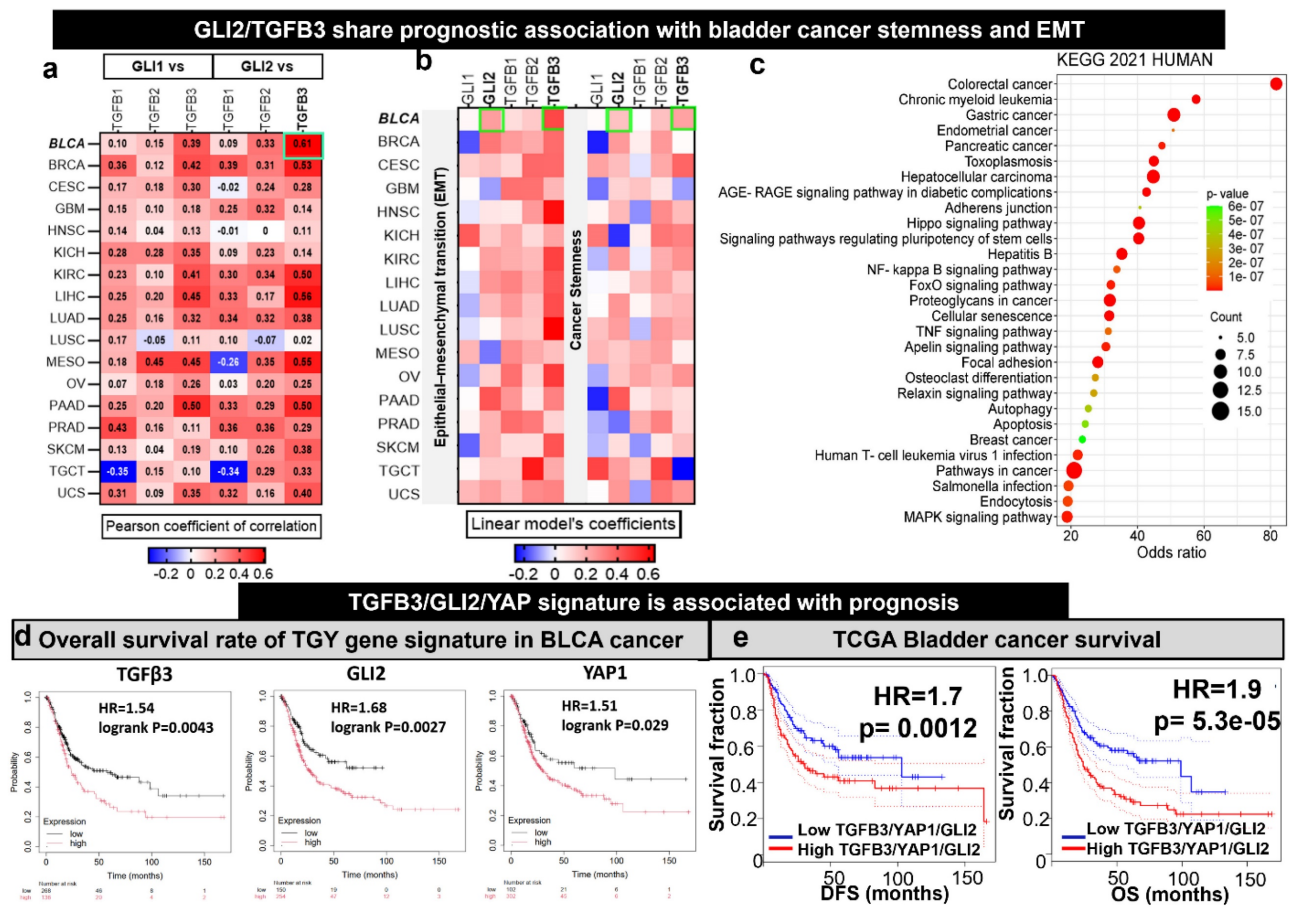
Increased TGFβ3/GLI2/YAP1 (TGY signature) signaling is predominately associated with EMT and stemness in bladder cancer patients. (**A**) A heatmap depicts the expression correlation between GLI1 and GLI2 and TGFβ3 genes in TCGA cancer types. Notably, TGFβ3 is expressed highest among the bladder cancer cohort's three TGFB family members (1, 2, and 3). (**B**) A heatmap represents the Pearson correlations between GLI2 and TGFβ3 and the signature of EMT/stemness in TCGA cohorts. (**C**) TGFβ3 gene enrichment analysis. The bubble plot shows the signaling and metabolic pathways curated from the KEGG database 2021. (**D**) Kaplan-Meier survival curves showing the expression of TGFβ3/GLI2/YAP1 in TCGA cohorts of bladder cancer. (**E**) Kaplan-Meier survival curves demonstrate that the higher expression of the TGY signature is associated with lower overall survival (right) and disease-free survival (left) of the TCGA bladder cancer cohorts.

We examined the expression correlations of GLI1/2 with TGFβ1/2/3 from the transcriptomic data of TCGA cohorts reported in a seminal study 15. GLI1 and GLI2 demonstrated a higher correlation with TGFβ3 than TGFβ1 and TGFB2 in all the TCGA cohorts (**Fig. 1A**). Notably, the GLI2 demonstrated a higher correlation (cor = 0.61, p<0.001) with TGFβ3 in bladder cancer when compared to other cancer type. Interestingly, the GLI2 and TGFβ3 share prognostic value with EMT and stemness signature in BRCA (**Fig. 1B**). This bladder cancer patient cohort consisted of 1154 samples and offered solid support for our hypothesis that TFGβ3 is a critical marker for bladder cancer progression and drug development. Next, we performed a gene-enrichment analysis based on TGFβ3 using the Pathway Commons Protein-Protein Interactions dataset (containing 42 interacting proteins for TGFβ3) 29. The results indicated that TGFβ3 is associated significantly with different cancer types and participates in many cancer signaling pathways, including hippo and proteoglycan signaling and pluripotent stem cell regulations (**Fig. 1C**). Since GLI2 and hippo signaling are well established to be involved in the generation and maintenance of cancer stem cells, we added YAP1, an established cancer stemness marker from the hippo pathway into the gene signature 44. In support, a survival analysis from the TCGA database indicated that elevated TGFβ3, GLI2, and YAP1 mRNA levels significantly correlated with an OS rate in bladder cancer patients (p < 0.05), high GLI2 expression decreased the outcome the most with HR of 1.68, TGFβ3 and YAP1 exhibited the HRs of 1.54 and 1.51, respectively (**Fig. 1D**). In addition, high expression of this trio was significantly associated with lower survival outcome (HR = 1.9, p < 0.001) and disease-free survival (HR = 1.7, p < 0.01 of the BLCA cohort (**Fig. 1E**). Collectively, the bioinformatics analysis identified TGFβ3/GLI2/YAP1 as a potential target gene signature (TGY signature) in bladder cancer.

### TGFβ3/GLI2/YAP1 signature is associated with an immunosuppressive tumor microenvironment and resistant to immunotherapy

**Figure 2:**
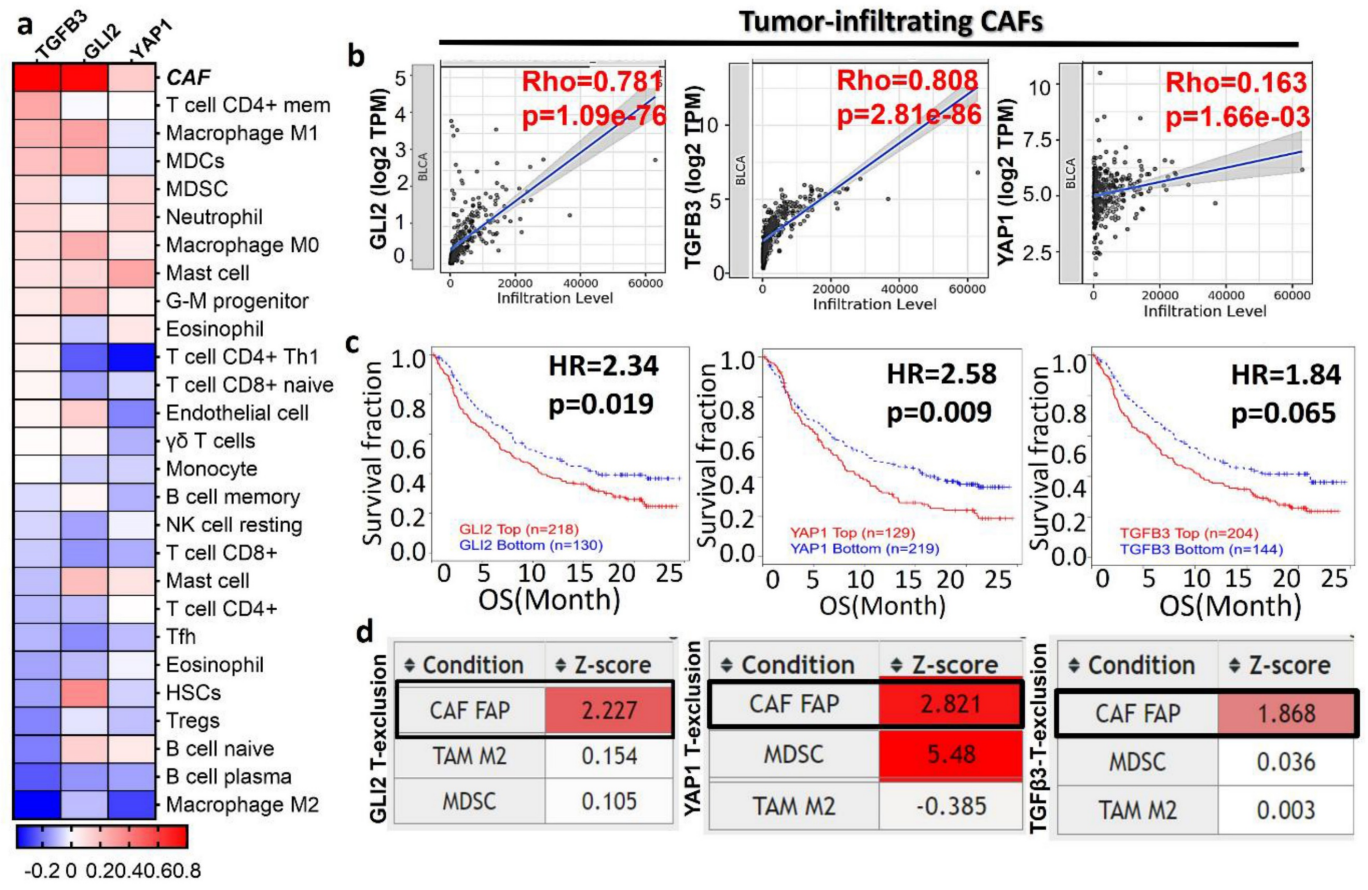
TGFβ3 expression patterns are associated with bladder cancer progression and immune-suppressive tumor microenvironment (TME). (**A**) TCGA database analyses revealed that TGY signature expression was significantly correlated with various immune cells and CAFs (p < 0.01). (**B**) CAF was found to be the predominant infiltrated immune cells. (C). High expression of the TGY signature statistically worsens the OS rate of mUC patients. (D). TGFβ3/GLI2/YAP1 was closely associated with the CAF-mediated T cell exclusion phenotype.

The tumor microenvironment (TME) plays a pivotal role in cancer progression through the facilitators, such as cancer-associated fibroblasts and immune cells (macrophages and T cells) within the TME 7. We examined the association of TGFβ3/GLI2/YAP1 with tumor infiltration of various immune and immunosuppressive cells (**Fig. 2A-B**). TGFβ3/GLI2/YAP1 expression was associated with anti-tumor immune desertion, as evident by a negative association with tumor-infiltrating lymphocytes, including the CD8 T cells, CD4 T cells, B cells, Th1, gamma delta T cells (γδ T cells), and T follicular helper cells (Tfh) (**Fig. 2A**). Notably, TGY signature showed the highest correlation with CAFs: TGFβ3 (r = 0.81, p = 2.81e-86), GLI2 (r = 0.78, p = 1.0e-76), and YAP1 (r = 0.16, p = 1.66e-03), respectively (**Fig. 2B**). We further analyzed the transcriptomic and clinical data from a large cohort of patients with mUC (metastatic urothelial carcinoma) treated with atezolizumab. Patients with high expression levels of TGFβ3/GLI2/YAP1 were more resistant to anti-PD1 treatment, exhibiting shorter OS; HR = 2.3, p = 0.019 (GLI2), HR = 2.6, p = 0.009 (YAP1), and HR = 1.84, p = 0.065, (TGFβ3) when compared to cohorts with low expression levels of this gene signature (**Fig. 2C**). Furthermore, this gene signature was associated with the CAF-mediated T cell exclusion phenotype of these patients (**Fig. 2D**). This phenotype is characterized by the restricted T lymphocyte cells infiltrating the tumor core, eventually contributing to tumor immune evasion. Collectively, our bioinformatics results strongly support that the TGFΒ3/GLI2/YAP1 expression level is significantly associated with tumor-infiltrating CAFs, T cell desertion, and immunosuppressive phenotypes and is resistant to immunotherapy in bladder cancer.

### Elevated TGFβ3, GLI2, and YAP1 expression in bladder tumorspheroids and CAF infiltration

**Figure 3:**
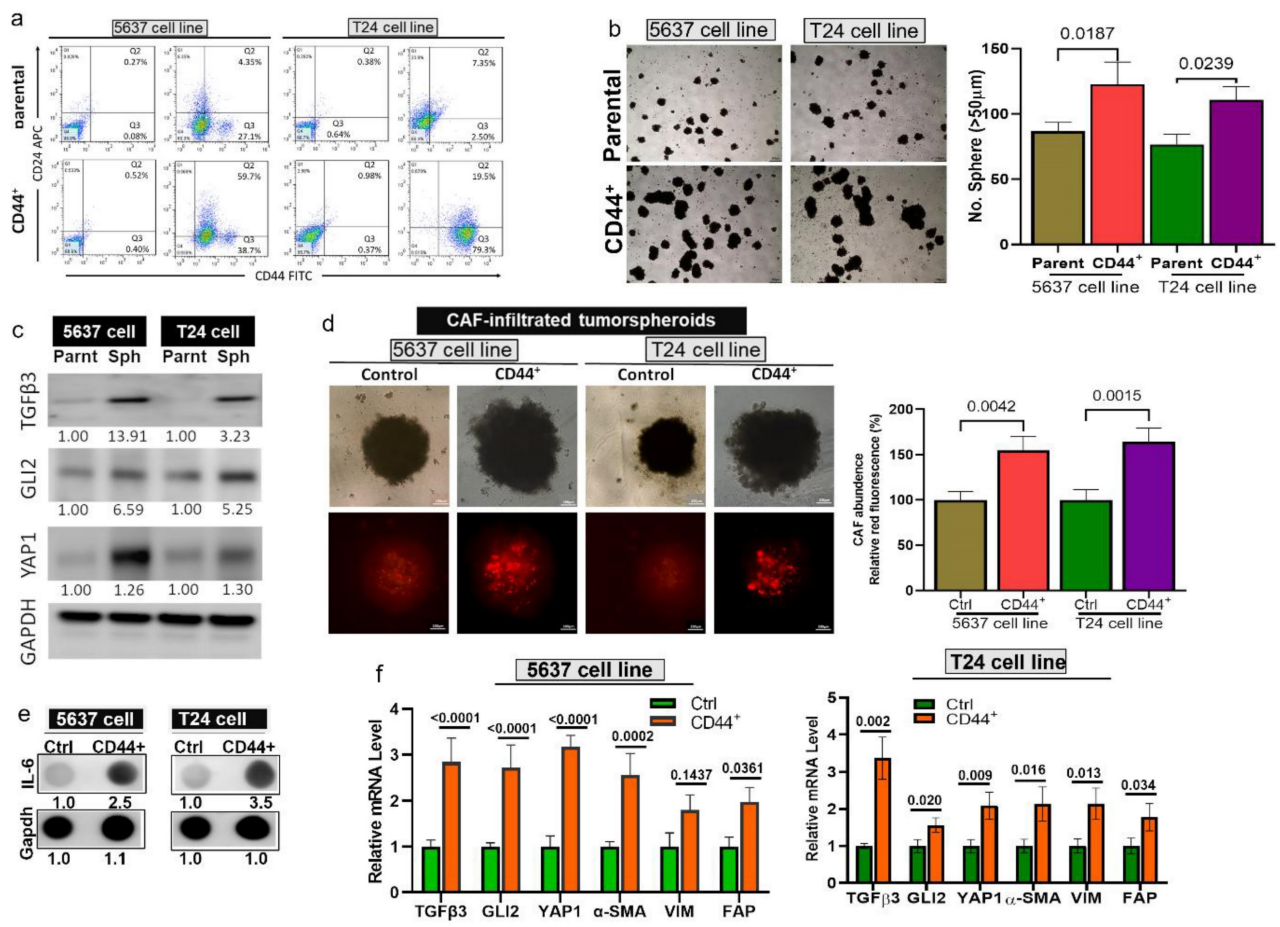
Elevated TGFβ3, GLI2, and YAP1 expression in bladder tumorspheroids and CAF infiltration. (**A**) Flow cytometric analysis demonstrates a higher % CD44 cells in the T24 and 5637 bladder tumorspheroids than their parental counterparts. (**B**) Representative images (left panel) of the tumor spheroid-generating ability of the CD44^+^ T24 and 5637 cells when compared with their parental counterpart; the right panel shows the spheroid quantification. (**C**) Comparative western blots show the upregulation of TGFβ3, GLI2, and YAP1 in CD44^+^ T24 and 5637 tumorspheroids compared with their parental counterparts. (**D**) Microscopic images of cancer-associated fibroblasts (CAF) infiltration levels between the CD44^+^ tumorspheroids and their parental counterparts. The intensity of the red fluorescence reflects the CAF infiltration. The fluorescence quantification is displayed in the bar graphs (right panel). (**E**) Dot blots of IL-6 secretion comparison between the CD44^+^ CAF-infiltrated tumorspheroids and their parental counterparts. (**F**) Quantitative PCR analysis comparing the mRNA levels of TGFβ3, GLI2, and YAP1, α-SMA (alpha-smooth muscle actin), vimentin, and FAP (Fibroblast activation protein) between the CD44+ CAF-infiltrated tumorspheroids than their parental counterparts.

Next, to test our hypothesis, we generated bladder tumorspheroids by isolating CD44^+^ T24 and 5637 cells (**Fig. 3A**). CD44^+^ bladder cells demonstrated a significantly higher ability to form tumorspheroids compared to their parental counterparts (**Fig. 3B**) and expressed a markedly higher level of TGFβ3, GLI2, and YAP1 (**Fig. 3C**). Subsequently, we co-cultured T24 and 5637 cells with CAF to form CAF-infiltrated tumorspheroids. We observed a significantly higher CAF-infiltrating incidence in the CD44^+^ tumorspheroids than their parental counterparts (**Fig. 3D**), as reflected by the intensity of the red fluorescence. A previous study shows that interleukin 6 (IL6) signaling is pivotal in maintaining the stem-like properties of bladder CSCs 45. In support, IL-6 secretion by the CD44^+^ CAF-infiltrated tumorspheroids was significantly higher than their parental counterparts, as demonstrated by the dot blots (**Fig. 3E**). Also, the mRNA levels of TGFβ3, GLI2, and YAP1 were significantly higher in the CD44^+^ CAF-infiltrated tumorspheroids as well as the CAF markers, α-SMA (alpha-smooth muscle actin), vimentin, and FAP (Fibroblast activation protein) (**Fig. 3F**).

### TGFβ3-silenced bladder cancer cells exhibited reduced tumorigenesis and CAF transformation

**Figure 4:**
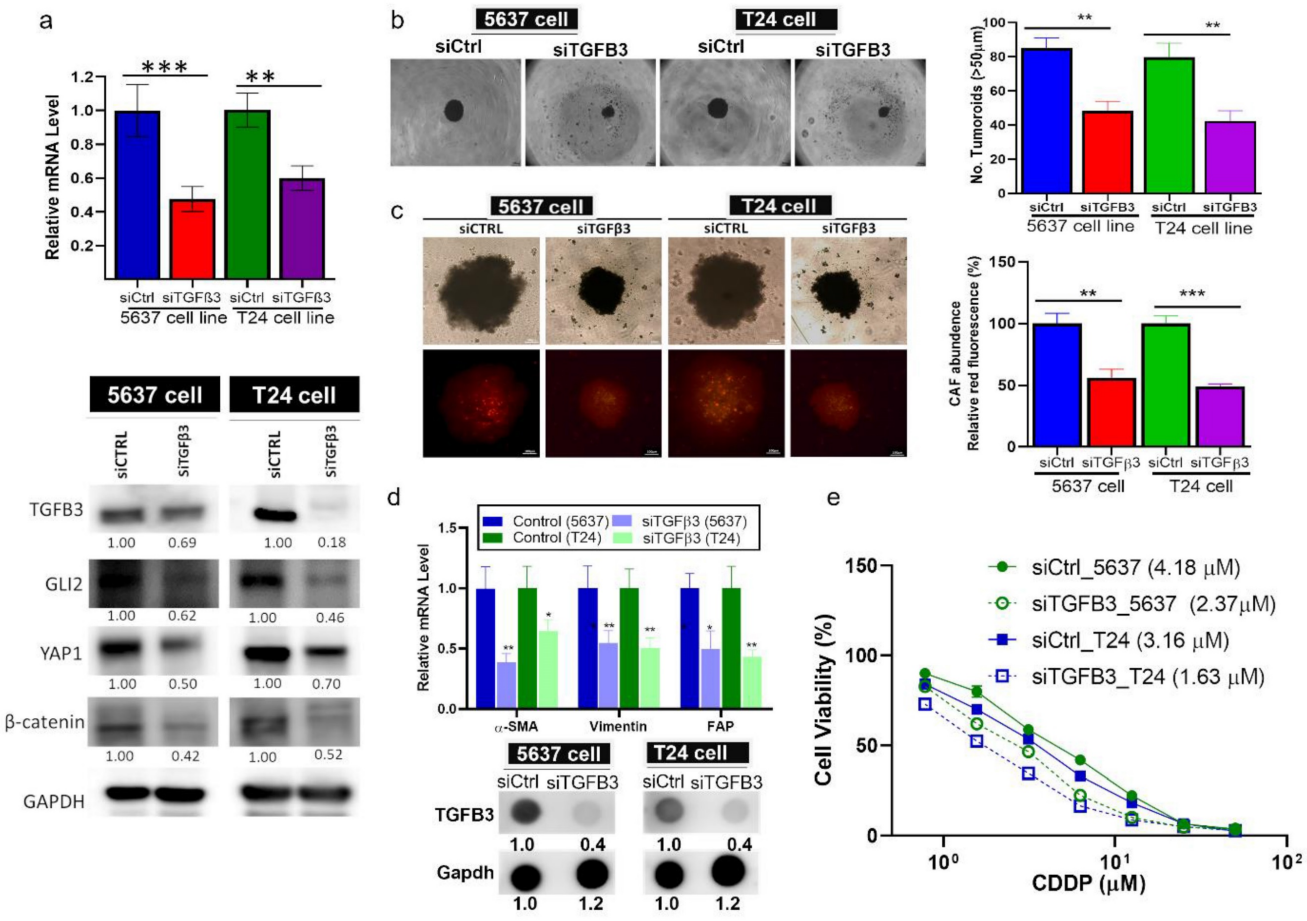
TGFβ3-silencing resulted in significantly reduced bladder tumorigenic properties. (**A**) Bar graph of mRNA expression levels confirming the TGFβ3-silencing effect of siRNA on T24 and 5637 cells. Western blotting shows the downregulation of TGFβ3, GLI2, YAP1, and β-catenin in TGFβ3-silenced T24 and 5637 cells compared with the siCtrl. Microscopic image (left panel) showing the (**B**) reduced tumorsphere generating ability, and (**C**) CAF-infiltration of TGFβ3-silenced T24 and 5637 cells when compared with the siCtrl: the left panel shows the quantification of the spheroid and CAF-infiltration respectively. (**D**) Bar graph showing the downregulation of the mRNA levels of α-SMA, vimentin, and FAP in the TGFβ3-silenced T24 and 5637 cells as compared with the siCtrl counterparts. The dot blot on the lower panel also shows decreased TGFβ3 secreted in the TGFβ3-silenced cells than their siCtrl counterparts. (**E**) A drug-response curve showing higher sensitivity of TGFβ3-silenced 5637 and T24 cells to cisplatin treatment than the siCtrl. Results are expressed as mean ± SD of assays performed 3 times in triplicate. *p < 0.05, **p < 0.01, ***p < 0.001.

Next, we performed gene-silencing experiments to demonstrate the pro-tumorigenic role of TGFβ3. TGFβ3-silenced T24 and 5637 cells appeared to express a markedly lower level of GLI2, YAP1 and β-catenin (**Fig. 4A**). TGFβ3-silenced bladder cancer cells demonstrated a significantly reduced self-renewal ability as reflected by the significantly low number of tumorspheroids generated compared to their control counterparts (**Fig. 4B**). We further observed that CAF-infiltration was reduced considerably in the TGFβ3-silenced tumorspheroids (lower fluorescent intensity, **Fig. 4C**). In support, CAF-markers, α-SMA, vimentin and FAP and secreted TGFβ3 were markedly reduced in the TGFβ3-silenced tumorspheroids (**Fig. 4D**). Functionally, TGFβ3-silenced 5637 and T24 cells became more sensitive to cisplatin treatment, reflected by the lowered IC50 values (IC50 values are shown in the parentheses, **Fig. 4E**).

### TGN treatment reduced bladder tumorigenic properties and CAF transformation

**Figure 5:**
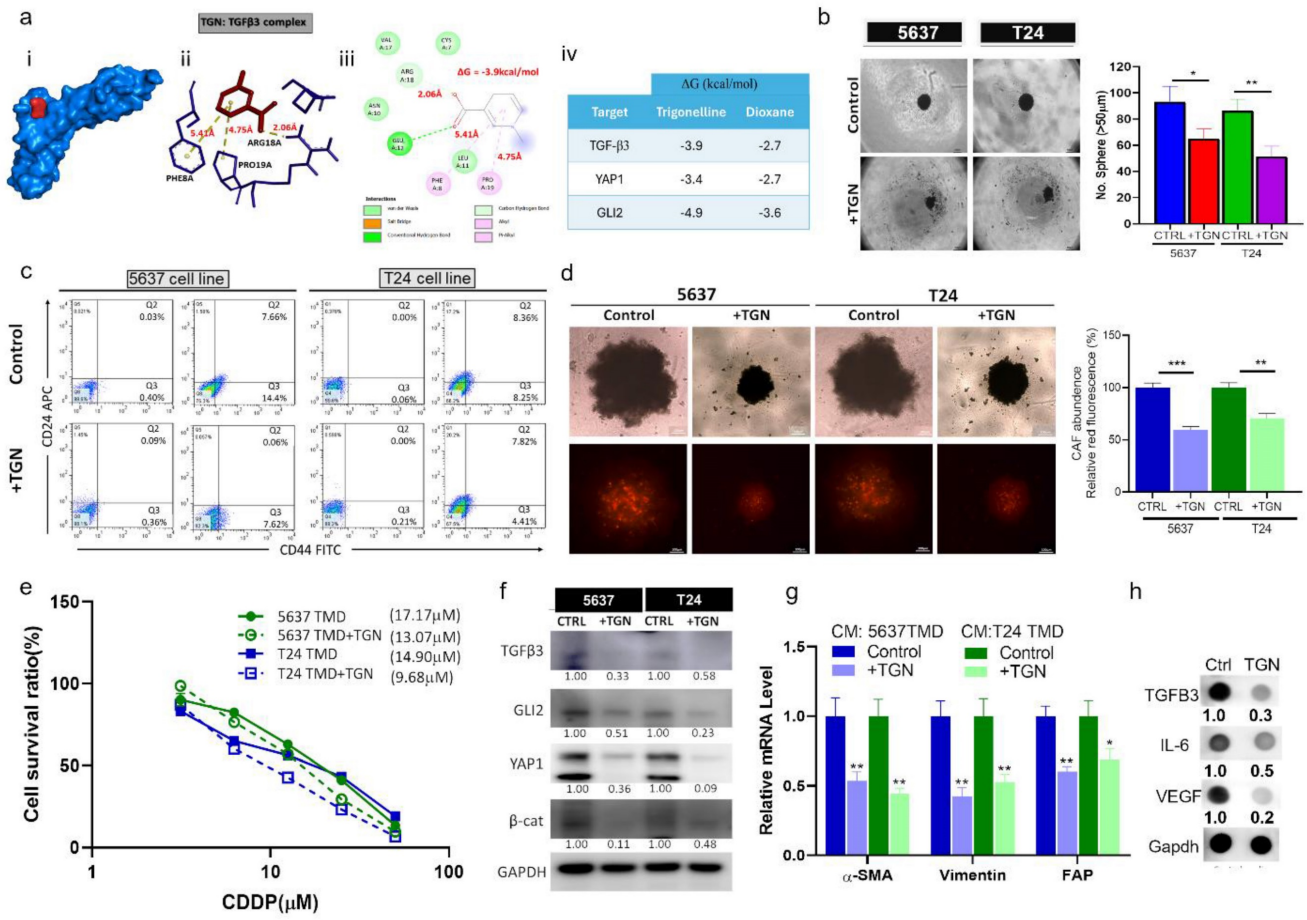
*In vitro* characterization of TGN's anticancer functions and CAF transformation. (A) Molecular docking simulation of TGN: TGFβ3 binding complex. (i) A 3D molecular complex demonstrates TGN (red) in TGFβ3's binding pocket. (ii) A stick illustration demonstrates the amino acid residues participating in the hydrophobic interactions within the pocket. The numbers represent the molecular distance of the bond. (iii) A 2D ligand interaction map of the TGN: TGFβ3 binding complex. This illustration shows the interacting amino acids, the type of interaction, and the bond distance. (iv) The table shows the binding energy (Gibb's free energy) calculated in each complex. (**B**) Effect of TGN on the tumor spheroid-forming ability of T24 and 5637 cells. The right panel represents a quantitative analysis of the spheroid formation between the TGN-treated (11 and 7 µM, 72 hs, for 5637 and T24 spheres, respectively) and the control cells. (**C**) Flow cytometric analysis shows the decreased percentages of CD44^+^ cells in TGN-treated T24 and 5637 cell lines compared to the control (non-treated) counterparts. (**D**) Representative micrographs demonstrating the TGN's inhibitory effects on CAF-infiltration in T24 and 5637 tumor spheroids (denoted as TMD). The bar graphs on the right represent the quantitative analysis of CAF infiltration levels between TGN-treated and control tumorspheroids. (**E**) The drug-response curve showed that TGN treatment increased the sensitivity of 5637 and T24 tumoroids to cisplatin treatment. (**F**) Western blots comparing the expressions of TGFβ3, GLI2, YAP1, and β-catenin in TGN-treated and control T24 and 5637 TMD. (**G**) Comparative qPCR analysis shows TGN-treated bladder tumorspheroids (TMD) expressed significantly lower mRNA expression levels of α-SMA, vimentin, and FAP than their control counterparts. (H) Comparative dot blot analysis of the culture media from control and TGN-treated tumoroids (11 and 7 µM, 72 hs, for 5637 and T24 TMD, respectively). TGFβ3, IL-6, and VEGF levels were markedly lower in the TGN-treated group. Results are expressed as mean ± SD of assays performed 3 times in triplicate. *p < 0.05, **p < 0.01, ***p < 0.001.

Based on our molecular docking simulations, TGN could form a stable complex with TGFβ3 as demonstrated by a free Gibb's energy of -3.9 kcal/mol (**Fig. 5A**). To our knowledge, there is currently no established TGFβ3 inhibitor. So we searched the Drug Bank database (https://go.drugbank.com/polypeptides/P10600) and found that 1,4-Dioxane was suggested as a potential inhibitor. Our molecular docking analysis demonstrated that TGN bound to TGFβ3 with a greater affinity (Gibb's free energy -3.9 versus -2.7 kcal/mol) than 1,4-Dioxane (**Fig. S2**). We then demonstrated that TGN treatment significantly reduced tumor spheroid-generating ability in both cell lines. TGN-treated T24 and 5637 cells formed a substantially lower number of tumorspheroids compared to their control counterparts (**Fig. 5B**). In addition, TGN treatment led to the decreased number of CD44^+^ T24 and 5637 cells according to our flowcytometric analysis (**Fig. 5C**). We then generated bladder tumoroids (containing CAFs) to assess TGN's therapeutic function. TGN treatment led to a significantly lower CAF transformation, as reflected by the lower red fluorescence intensity (**Fig. 5D**). Subsequently, we subjected TGN-treated bladder tumoroids (TMD) to cisplatin treatment. TGN-treated tumoroids were more sensitive to cisplatin than their control counterparts, as reflected by the lower IC50 values (**Fig. 5E**). Comparative western blots of control and TGN-treated tumoroids showed that TGN treatment reduced the expression of TGFβ3, GLI2, YAP1, and β-catenin (**Fig. 5F**). Furthermore, we examined the TGN-treated tumoroids' ability to transform normal fibroblasts to CAFs. Fibroblasts cultured with the conditional medium (CM) from TGN-treated 5637 and T24 tumoroids expressed significantly lower mRNA levels of α-SMA, vimentin, and FAP (**Fig. 5G**). CM from TGN-treated tumoroids consistently showed markedly reduced secretion of TGFβ3, IL-6, and VEGF (**Fig. 5H**).

### *In vivo* demonstration of TGN treatment overcame cisplatin resistance

**Figure 6:**
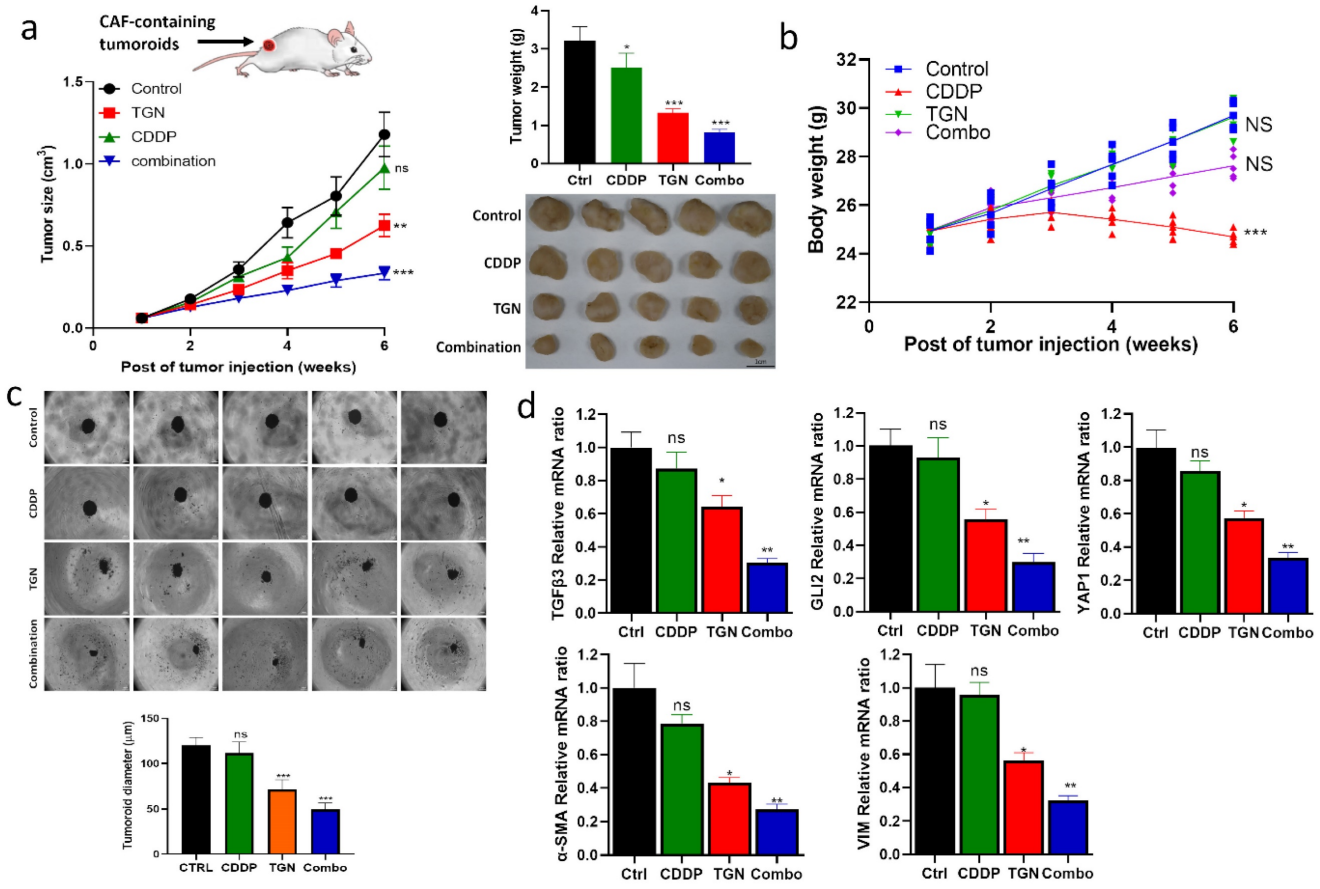
TGN treatment delayed tumoroid growth and overcame cisplatin resistance in a mouse xenograft model. (**A**) The tumor volume versus time graph demonstrates the effect of trigonelline (TGN) monotherapy, cisplatin (CDDP) monotherapy, and combination therapy in a CAF-containing 5637 tumoroid xenograft mouse model. Vehicle control (PBS), trigonelline monotherapy (50 mg/kg, 3 times/week, i.p), cisplatin monotherapy (1 mg/kg, 3 times/week, i.p), and combination therapy (TGN: 50 mg/kg, 3 times/week, i.p; cisplatin: 1 mg/kg, 5 times/week, i.p). N=5 for each group. The upper right panel shows the average tumor weight from each group; the lower right panels demonstrate the photos of the tumor samples harvested after experiments. (**B**) Mean body weight versus time graph. The body weight of the mice was recorded weekly. The average weights were then plotted against time for monitoring purposes. (**C**) Tumor spheroid forming assay. The secondary tumor spheroid-forming ability of the tumor cells harvested from each group was analyzed. The diameter represented the average size of the tumor spheroids. (**D**) Quantitative PCR analysis comparing the expression of TGFβ3/GLI2/YAP1 (the TGY signature) and CAF markers (α-SMA and vimentin) among different groups. Results are expressed as mean ± SD of assays performed 3 times in triplicate. **p < 0.01, ***p < 0.001, ****p < 0.0001.

After establishing the *in vitro* efficacy of TGN, we intended to evaluate its efficacy *in vivo*. We established a cisplatin-resistant xenograft model by subcutaneously injecting 5637 tumorspheroids (TMD) containing CAFs. We then subdivided the mice into four groups: control, cisplatin (CDDP) alone, TGN alone, and a combination of TGN and CDDP. The tumor growth curves demonstrated no significant difference in the overall tumor sizes in the control and CDDP groups. TGN treatment significantly delayed the tumor growth compared to the control and CDDP counterparts (**Fig. 6A**), while the combination treatment showed the most significant tumor growth delaying effect (**Fig. 6A-B**). We also observed that adding TGN protected against the cytotoxic effect of CDDP, as the combination group did not show a decrease in body weight over time (**Fig. 6B**). After the experiments, the tumor samples were harvested. We examined their ability to form tumorspheroids under serum-deprived culture conditions. We found that tumor cells harvested from the TGN and combination groups had the smallest tumorspheroids formed (**Fig. 6C**). We also examined the TGFβ3/GLI2/YAP1 (the TGY signature) expressions in the tumor samples. TGN and combination treatment groups consistently demonstrated significantly lower mRNA levels of the TGY signature. In contrast, the control and CDDP groups showed similar expression levels (upper panels, **Fig. 6D**). Similarly, CAF markers α-SMA and vimentin were significantly downregulated in TGN and the combination regimen samples (lower panels, **Fig. 6D**).

## Discussion

Trigonelline (TGN), a naturally occurring alkaloid predominantly isolated from coffee beans and fenugreek seeds, has gained much attention in the pharmacological and medical fields due to its diverse biological activities. TGN's therapeutic potential in oncology has been increasingly examined due to its potent antioxidant function. TGN has been demonstrated to inhibit the Nrf2 activity, a well-established signaling pathway that contributes to chemoresistance, by regulating antioxidants and detoxification enzymes. This inhibition has been shown to increase colon 46 and non-small cell lung cancer 47 cells' sensitivity toward platin-based chemotherapeutic agents. However, TGN's application in treating drug-resistant bladder cancer has not been well explored. In this study, we identified molecular candidates in drug-resistant bladder cancer cells and evaluated TGN as a potential inhibitor.

We started with a bioinformatics approach and identified a novel gene signature, TGFβ3/GLI2/YAP1 (the TGY) signature, significantly associated with immune evasion and therapy resistance in bladder cancer. An elevated TGY signature is associated with poor survival for bladder cancer patients. Individual members of the TGY signature have been previously shown to be oncogenic in different cancer types. However, their combined role as a signaling unit that promotes tumorigenesis in bladder cancer is a novel finding.

TGFβ3 is a less studied member of the TGF beta family. It has been shown to suppress and promote tumorigenesis depending on the disease stages 48, making it challenging to elucidate its precise role in bladder tumorigenesis. A recent study suggested that TGFβ3 was co-expressed with other hub genes to form an oncogenic network in bladder cancer 49. According to our bioinformatics results, TGFβ3 is also significantly expressed higher in the bladder cancer cells. More importantly, TGFβ3's expression significantly correlates with the level of tumor-infiltrating CAFs, suggesting it has dual roles in promoting tumorigenesis and shaping a pro-survival TME. Interestingly, a recent study using single-cell RNA sequencing technology reports that different subsets of fibroblasts express varying levels of TGF-β isoforms; a subset of myofibroblasts (myoFib-2) expressed a higher expression of TGFβ3 compared to other fibroblast subsets. The authors attributed this increased TGFβ3 expression in myoFib-2 to its role in activating CAFs and CAF-mediated extracellular matrix remodeling and tumor progression 50. Similarly, a previous study showed that human dermal fibroblasts (HDFs) with increased TGFβ3 expression enhanced the invasive properties and EMT of skin squamous cell carcinoma 51. Our observations showed that bladder tumorspheroids (generated from CD44^+^ cells) with increased TGY expression contained a higher percentage of CAFs and were accompanied by increased cisplatin resistance. The silencing of TGFβ3 resulted in reduced CAFs, further supporting TGFβ3's role in CAF transformation in the context of bladder cancer.

The second member of the TGY signature, GLI2, is a key transcription factor in the Hedgehog signaling pathway. GLI2 has been implicated in the maintenance of cancer stem cells and the promotion of EMT in various cancers 52. From a pan-cancer large-scale analysis 15, we could further extrapolate that GLI2 is most significantly associated with TGFβ3 in bladder cancer patients, laying the foundation for this study. Additionally, we found that GLI2 expression significantly correlates with the level of tumor-infiltrating CAFs, second to TGFβ3, providing further support for targeting this signaling axis as a therapeutic target.

The third member of the TGY gene signature, YAP1, was selected based on the gene set enrichment analysis where the TGFβ3/GLI2 gene set was significantly associated with the Hippo signaling pathway; in support, YAP1 was shown to induce GLI2 expression in promoting invasive phenotype in breast cancer patients 53. YAP1 was also shown to participate in EMT and maintain hepatocyte self-renewal ability by interacting with TGFβ signaling 54. Thus, these findings establish connections between YAP1 and the TGFβ3/GLI2 signaling. Although YAP1 expression was least significantly correlated with the level of tumor-infiltrating CAFs, its expression in the bladder cancer cohort remained predicted for poor survival. These findings implicated that the high expression of TGFβ3 and GLI2 could be responsible for recruiting and transforming CAFs. In contrast, YAP1 expression is associated with maintaining the stemness of bladder cancer cells.

Cancer-associated fibroblasts (CAFs) have been shown to contribute to drug resistance in different cancer types, including bladder 55, 56. However, the molecular link between the tumor cells and CAFs remains underappreciated. Identifying the TGY signature linking the bladder cancer cells with CAFs provided a clear molecular target for therapeutic development. This is supported by our gene silencing data, where TGFβ3-silenced bladder cancer cells exhibited a markedly reduced ability to generate CAFs and tumorspheroids (stemness and drug resistance). In addition, we demonstrated that TGN, a phytochemical, formed a stable complex with TGFβ3 in molecular docking simulations. TGN was selected as a drug candidate for this study based on its inhibitory effects on inflammation, oxidation, and angiogenesis 57. More importantly, a previous study demonstrated that TGN treatment inhibited EMT and subsequent fibrosis in a renal fibrosis model 58. However, TGN's function as an anticancer and TME agent was not explored. Our results provided evidence that TGN treatment significantly reduced bladder tumorigenic properties, notably tumor spheroid forming ability, cisplatin resistance, and CAF transforming capability; TGN's anticancer function was associated with the downregulation of the TGY signature, supporting our observations where TGFβ3 plays a key role in CAF transformation. Furthermore, TGN treatment reduced the CAF-containing tumoroids' production of TGFβ3, IL-6 59, and VEGF 60, all promoting angiogenesis, drug resistance, and metastasis.

In summary, our study identifies a unique TGFβ3/GLI2/YAP1 (the TGY signature) that is not only elevated in bladder cancer cells but also plays a crucial role in CAF transformation and function. Our data establishes a novel molecular link between cancer cell intrinsic signaling and the tumor microenvironment. It offers a potential explanation for the coordinated progression of bladder tumors and their surrounding stroma. Finally, TGN was shown to reduce cisplatin resistance CAF transformation and ultimately delay the progression of bladder cancer cells. Targeting the TGY signature with TGN represents a promising approach to address multiple aspects of bladder cancer tumorigenesis, potentially leading to more effective and personalized treatment strategies for patients with this clinical challenge.

## Supplementary Material

Supplementary figures and tables.

### Funding

Bashir Lawal is supported by the Hillman Postdoctoral Fellowship for Innovative Cancer Research award. Alexander TH Wu is supported by the National Science and Technology Council (113-2314-B-038-010 and 113-2320-B-038-034). Jing-Wen Shih and Ming-Che Liu were supported by the Taipei Medical University Hospital Research Grant (112TMU-TMUH-04). Ming-Che Liu is also supported by the National Science and Technology Council (113-2314-B-038 -107).

### Author contributions

Jing-Wen Shih, Alexander TH Wu, and Ming-Che Liu: Conceptualization and design. Bashir Lawal, Huong Thi Luu Kim Huynh, Chien-Chang Kao, Sitthichai Iamsaard, Po-Yang Huang, Nur Azizah and Ritmaleni Ritmaleni: Data acquisition and analysis. Bashir Lawal and Alexander TH Wu: Manuscript Drafting. Ming-Che Liu, Jing-Wen Shih, Ching-Hsin Chang, Justin Kung-Yi Lin, and Alexander TH Wu: Writing, review, and editing**.**

### Data availability statement

The datasets used and/or analyzed are available from the corresponding author upon reasonable request.

## Abbreviations

2D: two-dimensional
3D: three-dimensional, BLCA: bladder cancer
CAFs: cancer-associated fibroblasts
CDDP: cisplatin
CM: the conditional medium
CSCs: cancer stem cells
EMT: epithelial-mesenchymal transition
FACS: fluorescence-activated cell sorting
FAP: fibroblast activation protein
HDFs: human dermal fibroblasts
mUC: metastatic urothelial carcinoma
NMP: N-methylpyridinium iodide
OS: overall survival
PCR: polymerase chain reaction
RT: reverse-transcribed
SCR: scrambled
SDS-PAGE: sulfate-polyacrylamide gel electrophoresis
TAMs: tumor-associated macrophages
TGFβ: transforming growth factor-β
TGN: trigonelline
TMD: TGN-treated bladder tumoroids
TME: tumor microenvironment
Treg: regulatory T
α-SMA: alpha-smooth muscle actin
