# Supplementary material for: Nutraceutical Evaluation of Trigonelline's Therapeutic Potential by Targeting Bladder Cancer Stem Cells and Cancer-Associated Fibroblasts via Downregulation of TGFβ3/GLI2/YAP1 Signaling Hub
Source: Int J Med Sci. 2025 Feb 18;22(5):1194–207. doi: 10.7150/ijms.107228 (PMC11866525; doi:10.7150/ijms.107228)
Supplement: Supplementary file 1 — Supplementary figures and tables. [file ijmsv22p1194s1.pdf]

Nutraceutical Evaluation of Trigonelline's Therapeutic Potential By Targeting Bladder Cancer Stem Cells And Cancer-Associated Fibroblasts Via Downregulation Of TGFβ3/GLI2/YAP1 Signaling Hub

SUPPLEMENTARY FIGURES

Chien-Chang Kao[1†], Jing-Wen Shih[2,3,4†], Huong Thi Luu Kim Huynh[5], Ching-Hsin Chang[6,7,8], Bashir Lawal[9], Sitthichai Iamsaard[10,11], Nur Azizah[12,13], Ritmaleni Ritmaleni[12,13], Justin Kung-Yi Lin[14], Po-Yang Huang[15], Alexander T.H Wu[5,15-17]* and Ming-Che Liu[7,19-21]*

1. Division of Urology, Department of Surgery, Tri-Service General Hospital, National Defense Medical Center, Taipei, Taiwan. Email: guman2011@gmail.com
2. Ph.D. Program for Cancer Molecular Biology and Drug Discovery, College of Medical Science and Technology, Taipei Medical University and Academia Sinica, Taipei 11031, Taiwan.
3. Graduate Institute of Cancer Biology and Drug Discovery, College of Medical Science and Technology, Taipei Medical University, Taipei 11031, Taiwan.
4. TMU Research Center of Cancer Translational Medicine, Taipei Medical University, Taipei 11031, Taiwan. Email: shihjw@tmu.edu.tw
5. International PhD Program for Translational Science, College of Medical Science and Technology, Taipei Medical University, Taipei 11031, Taiwan
6. Institute of Microbiology and Immunology, National Yang-Ming Chiao Tung University, Hsinchu 30010, Taiwan.
7. Institute of Microbiology and Immunology, National Yang-Ming University, Taipei, 11221, Taiwan
8. TMU Research Center of Urology and Kidney, Taipei Medical University, Taipei, 11031, Taiwan. Email: jossp1029@gmail.com
9. UPMC Hillman Cancer Center, University of Pittsburgh, USA. Email: bal140@pitt.edu
10. Department of Anatomy, Faculty of Medicine, Khon Kaen University, Khon Kaen 40002, Thailand.
11. Research Institute for Human High Performance and Health Promotion, Khon Kaen University, Khon Kaen 40002, Thailand. Email: sittia@kku.ac.th
12. Laboratory of Medicinal Chemistry, Department of Pharmaceutical Chemistry, Faculty of Pharmacy, Universitas Gadjah Mada, Jl. Sekip Utara,Yogyakarta, 55281, Indonesia.
13. Curcumin Research Center, Faculty of Pharmacy, Universitas Gadjah Mada, Jl. Sekip Utara, Yogyakarta, 55281, Indonesia. Email: nurazizah1999@mail.ugm.ac.id Email: ritmaleni@ugm.ac.id
14. SPs Traditional Medical Clinic, Taipei, Taiwan. drjustinlin@ntu.edu.tw
15. The Ph.D. Program of Translational Medicine, College of Medical Science and Technology, Taipei Medical University, Taipei, 11031, Taiwan. Email: boyang0321@gmail.com
16. Clinical Research Center, Taipei Medical University Hospital, Taipei Medical University, Taipei 11031, Taiwan
17. Graduate Institute of Medical Sciences, National Defense Medical Center, Taipei 11490, Taiwan
18. Taipei Heart Institute, Taipei Medical University, Taipei 11031, Taiwan.
19. School of Dental Technology, College of Oral Medicine, Taipei Medical University Taipei 11031, Taiwan.
20. Department of Urology, Taipei Medical University Hospital Taipei 11031, Taiwan.
21. Graduate Institute of Clinical Medicine, School of Medicine, College of Medicine, Taipei Medical University Taipei 11031, Taiwan.

[†] These authors contributed equally
*Corresponding authors**:** Alexander TH Wu Email: chaw1211@tmu.edu.tw and Ming-Che Liu Email: d204097002@tmu.edu.tw

Table S1. List of primer sequences used for q-PCR experiments in this study.

Gene target	Forward	Reverse
Actin	TTGTTACAGGAAGTCCCTTGCC	ATGCTATCACCTCCCCTGTGTG
YAP1	TAGCCCTGCGTAGCCAGTTA	TCATGCTTAGTCCACTGTCTGT
GLI2	GGTACCATTACGAGCCTCATTC	AAAGCCGGATCAAGGAGATG
TGFb3	CTAAGCGGAATGAGCAGAGGATC	TCTCAACAGCCACTCACGCACA
Vimentin	TGGCACGTCTTGACCTTGAA	GGTCATCGTGATGCTGAGAA
FAP	GGAAGTGCCTGTTCCAGCAATG	TGTCTGCCAGTCTTCCCTGAAG
α-SMA	CTATGCCTCTGGACGCACAACT	CAGATCCAGACGCATGATGGCA

Table S2. List of antibodies used for Western blot analysis in this study.

Target	Dilution	Company and Catalog No	Predicted MW (kDA)
β - catenin	1:1000	Cell Signaling, β –catenin (6B3) Rabbit mAb, #9582	92
YAP1	1:1000	Proteintech, YAP1 Antibody, Rabbit Polyclonal, 13584-1-AP	70
GLI2	1:1000	Affinity Biosciences, GLI2 Antibody, Rabbit Polyclonal, DF7541	167
TGFb3	1:1000	Proteintech, TGF Beta 3 Antibody, Rabbit Polyclonal, 18942-1-AP	45-50
GAPDH	1:1000	Proteintech, GAPDH Antibody, Rabbit pAb, 10494-1-AP	36

Supplementary Figure S1.

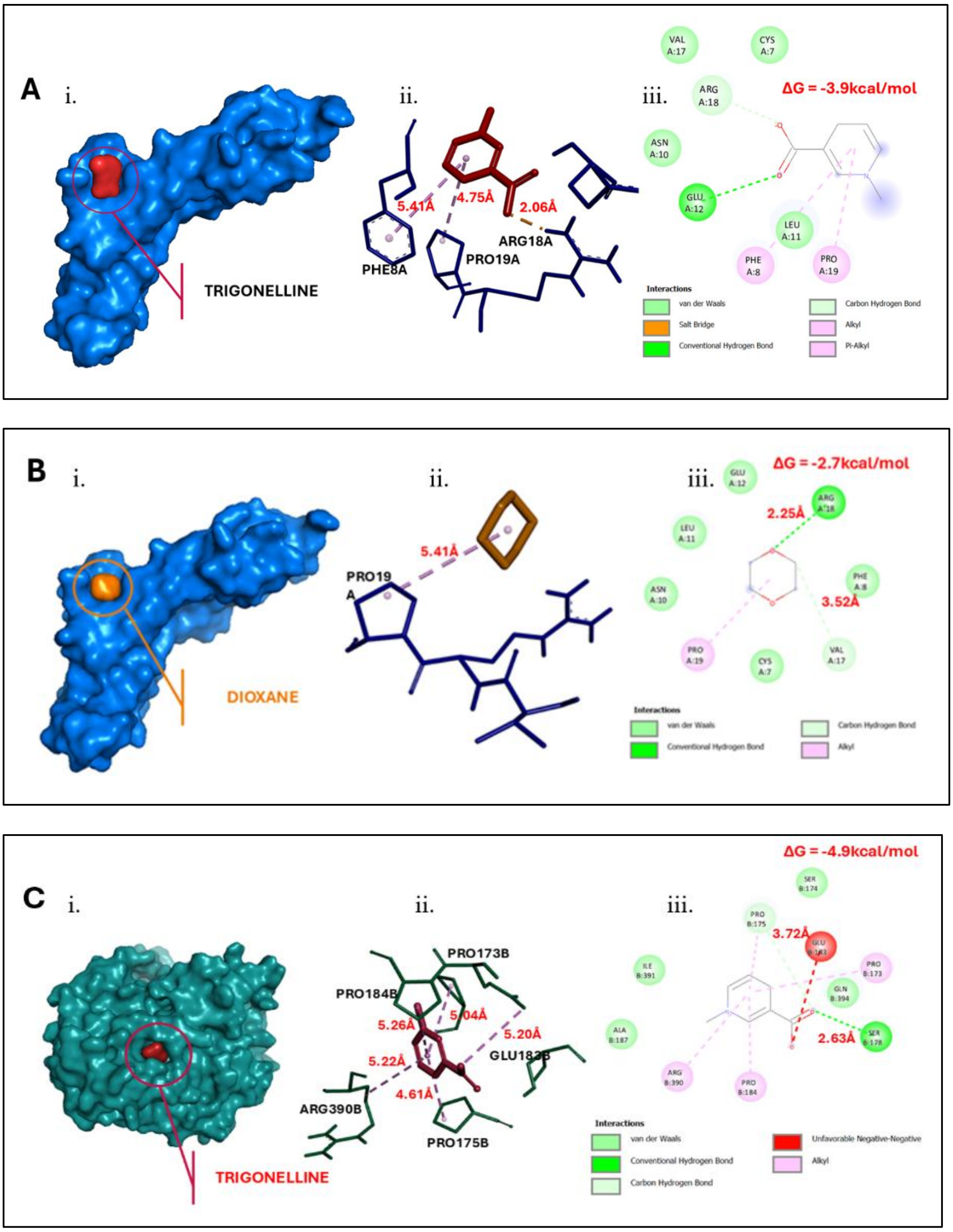

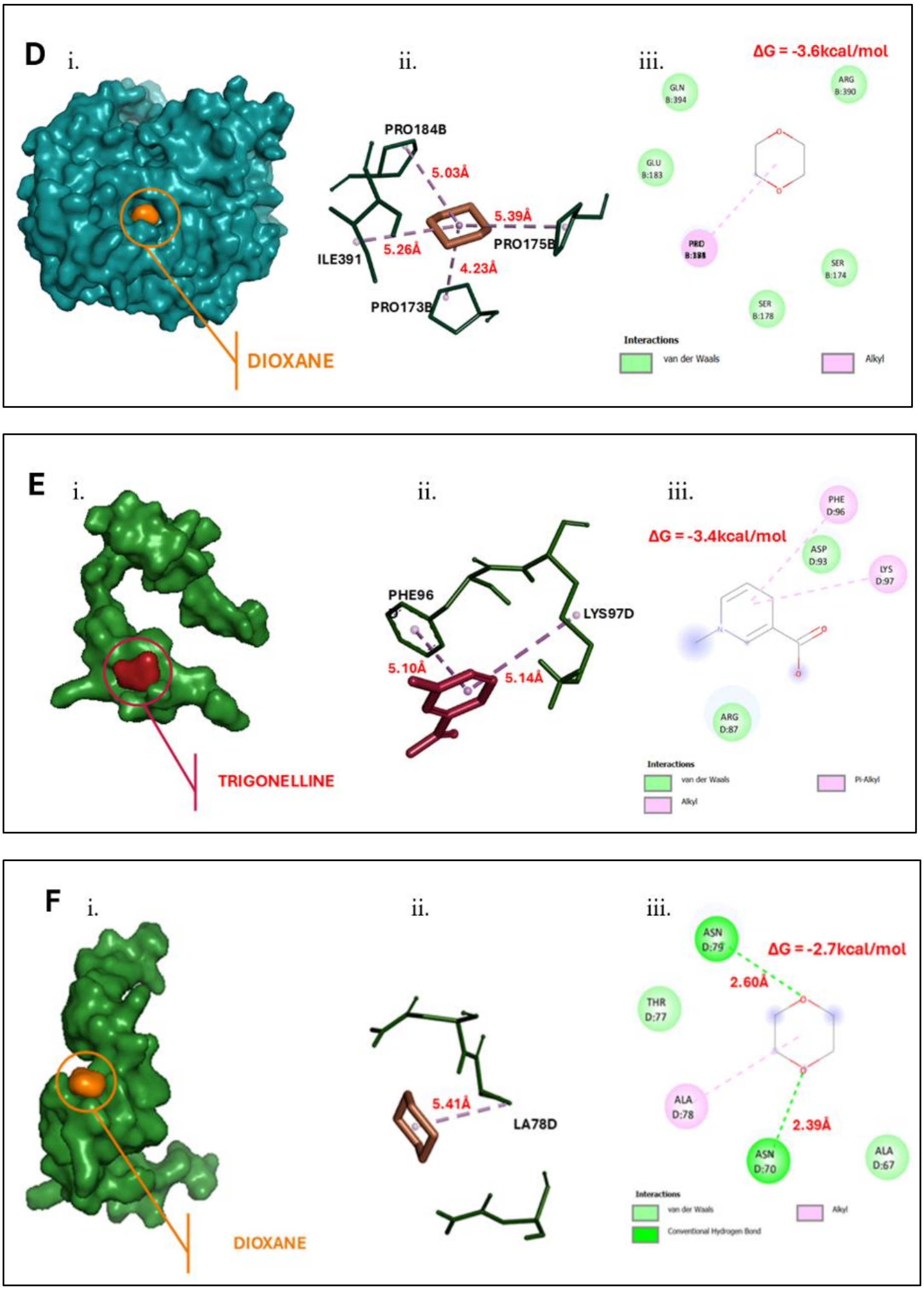

Figure S2. Molecular docking of Trigonelline, Dioxane (standard drug) bound to TGFβ3, GLI2, YAP1.

A, B: ***Trigonelline and dioxane docked to TGFβ3***. Trigonelline - TGFβ3 complex was stabilized with two hydrogen bonds, two alkyl bonds, one salt bridge, and Van der Waals. Their ΔG energy was higher than dioxane-TGFβ3 complex. **C, D**: ***Trigonelline and dioxane bound to GLI2***. Trigonelline docked to GLI2 with two hydrogen bonds and five alkyl bonds with the affinity binding was -4.9kcal/mol while the stability of

dioxane_GLI2 complex formed solely alkyl bonds and lower binding energy. **E, F**: ***Trigonelline and dioxane docked to YAP1***. Although dioxane targeted YAP1 with two hydrogen bonds, the affinity binding of this complex was lower than the ΔG energy of the trigonelline-YAP1 complex, which stabilized by alkyl interaction and Van der Waals.

***i**: 3D conformation presenting the binding pocket between the target gene and drug, **ii**: stick illustration showing the salt bridge (orange dash line), hydrophobic interactions between atoms and drug (pink dash line) and corresponding distance, **iii**: 2D diagram illustrating binding free energy, the Hydrogen interaction and corresponding distance (dash green line).
